# Laryngeal adenoid cystic carcinoma: Three cases reports

**DOI:** 10.1097/MD.0000000000018177

**Published:** 2019-12-20

**Authors:** Yu Cui, Lirong Bi, Le Sun, Xin Wang, Zhanpeng Zhu

**Affiliations:** a Department of Otolaryngology; b Department of Pathology; c Department of Neurosurgery, The First Hospital of Jilin University, Changchun, Jilin, P.R. China.

**Keywords:** adenoid cystic carcinoma, follow-up, laryngectomy, larynx, malignant tumors

## Abstract

**Introduction:**

Laryngeal adenoid cystic carcinoma (LACC) is an extremely rare malignant neoplasm. The etiology of LACC remains unknown, and it is characterized by multiple recurrences, slow progression, and late distant metastasis. This study aimed to provide more information regarding the characteristics, diagnosis, and treatment of LACC by analyzing 3 clinical cases and reviewing the literature on this topic.

**Patient concerns:**

Here, we present all 3 cases of LACC within the period between 2010 and 2019. Dyspnea was the most commonly observed symptom in these patients, followed by hoarseness, pharyngeal paresthesia, and difficulty swallowing.

**Diagnosis:**

All patients were pathologically confirmed as LACC.

**Interventions:**

All the patients underwent a combined therapy of surgical resection plus external irradiation.

**Outcomes:**

The follow-up time was between 2 and 6 years; no local recurrence occurred in any of the 3 patients. Lung metastasis was found in 1 patient 6 years after surgery.

**Conclusion:**

LACC is usually a slowly progressing cancer; the main treatment methods are surgery and radiotherapy, and the adequate radiotherapy dose should usually be greater than 60 Gy. The 5-year disease-specific survival rate is high; however, distant metastasis may still occur in patients with LACC even beyond 5 years after treatment. Therefore, patients with LACC require long-term surveillance.

## 1 Introduction

Adenoid cystic carcinomas, first described by Robin and Laboulbene in 1853, were previously known as cylindromas.^[1]^ This type of carcinoma mostly occurs in the minor salivary glands, which account for 1% to 5% of all head and neck malignancies.^[2–4]^ The common sites of adenoid cystic carcinomas are in the salivary glands of the oral cavity, in which the laryngeal adenoid cystic carcinomas (LACC) are sporadic, and account for less than 1% of laryngeal malignancy.^[5,6]^

The etiology of LACC remains unknown. It has no capsule and grows slowly, but has the ability to invade into surrounding tissues and distant metastasis. It is characterized by neurotropic growth, easy recurrence, and high rate of hematogenous metastasis,^[4]^ with no obvious association with smoking and drinking. Patients with LACC have a long survival period. However, multiple recurrences and late metastasis have been observed in this population. Thus, long-term follow-up is required.^[7]^

As LACC is rare, this study aimed to provide more information regarding the characteristics, diagnosis, and treatment of this disease by analyzing 3 clinical cases and reviewing the literature on this topic. This study was approved by the Jilin University Ethics Review Board.

## 2 Cases report

**Figure 1:**
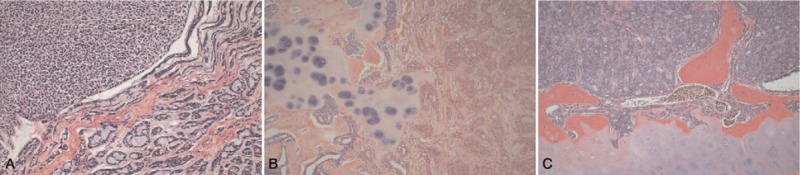
Imaging of microscope with laryngeal adenoid cystic carcinoma. (A) Solid pattern on the left side and tubular pattern on the right side (H&E staining, ×200). (B) Invasion of thyroid cartilage with ossification by cribriform pattern and tubular pattern (H&E staining, ×100). (C) Solid pattern adenoid cystic carcinoma invades thyroid cartilage with cartilage ossification (H&E staining, ×200).

We retrospectively analyzed 3 cases of LACC that were admitted to the First Hospital of Jilin University between 2010 and 2019. The clinical and pathological data were extracted from hospital medical records. We obtained follow-up information by contacting the patients or their family members. All the 3 patients have provided informed consent for publication of the case. The basic characteristics and treatment information of the patients are shown in Table 1. The follow-up data of the patients are shown in Table 2. The final pathological diagnosis in all cases was mixed LACC (see details in Fig. 1 and Table 3), and the proportion of subtypes are shown in Table 3.

**Table 1:**
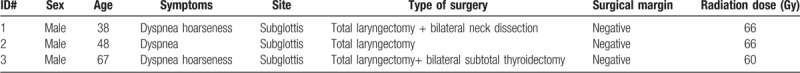
Patients’ basic characteristics and therapy.

**Table 2:**
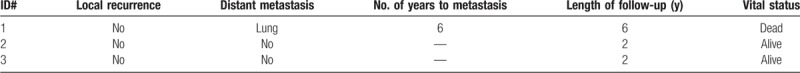
follow-up information for all three cases.

**Table 3:**
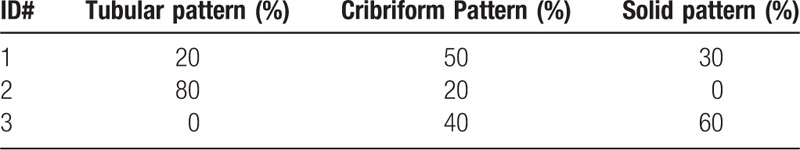
Histopathological patterns and the proportion.

### 2.1 Case 1

Case 1 was a male patient, 38 years old, who was admitted to hospital due to hoarseness accompanied by dyspnea after activities. Physical examination showed annular neoplasm in the subglottic portion, with subglottic stenosis; bilateral vocal cord movement was acceptable, and the neck ultrasound showed enlarged lymph nodes in the left neck. Intraoperative frozen section showed LACC. Therefore, total laryngectomy and left neck dissection were performed. The pathological margin was negative. Clinical diagnosis is LACC (T2N2bM0). Radiotherapy was accepted, and the radiotherapy dose was 66 Gy. Pulmonary metastasis was found 6 years after surgery, and the patient had died.

### 2.2 Case 2

Case 2 was a male patient, 48 years old, who was admitted to hospital due to laborious breathing and foreign body sensation in pharynx. Physical examination showed annular neoplasm in the subglottic portion, and no obviously enlarged lymph nodes in the neck. The laryngeal computed tomography showed irregular soft tissue shadows in the cricoid cartilage level. Intraoperative frozen section showed LACC, so total laryngectomy was performed. Clinical diagnosis is LACC (T2N0M0). Postoperative pathological margin was negative, radiotherapy was accepted, and the radiotherapy dose was 66 Gy. The follow-up period was 2 years, and the patient was still alive without distant metastasis.

### 2.3 Case 3

**Figure 2:**
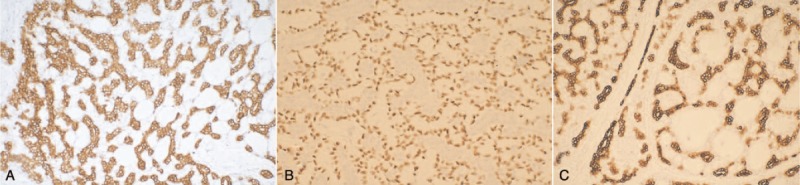
Imaging of immunohistochemical results of case 3: positivity of CD117 (H&E staining, ×200) (A), P63 (H&E staining, ×400) (B), and CK7 (H&E staining, ×200) (C).

Case 3 was a male patient, 67 years old, admitted to hospital due to laborious breathing and hoarseness. Physical examination showed annular neoplasm in the subglottic portion, no obviously enlarged lymph nodes in the neck, and the neck ultrasound showed multiple thyroid nodules. Intraoperative frozen section showed LACC and nodular goiter. Therefore, total laryngectomy and bilateral subtotal thyroidectomy were performed. Clinical diagnosis is LACC (T2N0M0)—nodular goiter. Immunohistochemical analysis of the specimen supported the diagnosis of LACC, and demonstrated positivity of CD117, P63, and CK7 (Fig. 2), which suggested that the tumor consisted of glandular epithelium and myoepithelium. The diagnosis of the other 2 cases is arrived at similar immunohistochemical result. Postoperative pathological margin was negative, radiotherapy was accepted, and the radiotherapy dose was 60 Gy. The follow-up period was 2 years. The patient was still alive without distant metastasis.

## 3 Discussion

Adenoid cystic carcinoma originates from large or small salivary glands or mucinous glands of the upper respiratory tract. The salivary glands of the larynx are very rare. Therefore, LACC is relatively rare.^[8]^ The distribution of the disease has been reported to gradually reduce from the supraglottic area to the glottis area and the subglottic area.^[8]^ Ellington et al^[9]^ found that the most common primary site of LACC is indeed the subglottic region (58.2%), followed by the upper glottic area (32.1%) and the glottic area (9.7%). In the majority of cases, the age of disease onset is between 50 and 60 years old.^[9,10]^ Although it is rare that patients with LACC are under 20 years of age, Javadi et al reported a case of a 12-year-old boy with subglottic adenoid cystic carcinoma.^[11,12]^ The symptoms and signs of LACC are closely related to the anatomical location of the tumor. Dyspnea is often associated with subglottic tumors, while glottic tumors are always characterized by pharyngeal paresthesia.^[13]^ In the present study, the location site of all 3 cases was in the subglottic region with a common symptom of dyspnea.

The clinical features of LACC include a high rate of neurological involvement,^[2]^ local invasive growth (in addition to nerve, bone, lymphatic vessels, blood vessels, and muscle involvement), a high recurrence rate, and a low rate of cervical lymph node metastasis (depending on the primary site of the tumor, the oral ACC lymph node metastasis rate can reach 37%).^[3,14]^ This condition is prone to distant metastasis, and even early lesions have the potential for distant metastasis. The most common metastatic organ is the lung, followed by bone and liver, and distant metastasis can still occur after several decades. Imaging evaluation at the time of follow-up is recommended.^[4,13]^ Because of the low incidence, atypical symptoms, and late onset of symptoms, detection of LACC is not easy. Thus, LACC is always in the advanced stage at the time of diagnosis. In addition, LACC has an “inert” biological behavior with a longer survival period. Even local recurrence or distant metastasis (especially in the lungs) can occur after a long period of treatment.^[15]^ Long-term follow-up and intervention are suggested after treatment. In 1 of the present cases, lung metastasis was observed after 6 years of treatment.

Adenoid cystic carcinoma is a basal cell-like malignant tumor that is composed of epithelial and myoepithelial cells. The histopathological classification of this carcinoma can be divided into 3 patterns: tubular, cribriform, and solid. The most commonly observed pattern is the solid type, and the prognosis of this pattern is poor.^[16–18]^ The 3 pathological patterns listed above may occur in the same case.^[18]^ One of the classification methods is performed according to the percentage of solid components, which is very convenient for prognosis prediction.^[19]^ All 3 of the cases included in this study belonged to mixed adenoid cystic carcinoma (Fig. 1). It is notable that in the patient in which pulmonary metastases were observed after 6 years of treatment, the cribriform pattern was predominant. Although the patient with pulmonary metastases was predominantly cribriform pattern, not solid pattern, as the number of cases was small, it did not contradict the results reported in the previous literature.

At present, surgery is the most successful method used for the treatment of LACC. It remains a controversial issue whether it is necessary to perform adjuvant radiotherapy or chemotherapy. The majority of scholars believe that adjuvant radiotherapy (mainly postoperative radiotherapy) is needed, and this is recommended as the standard treatment model, which is considered to be superior to surgery alone or radiotherapy alone.^[4,16]^ For LACC in the subglottic region, a total laryngectomy is required, which was performed in all 3 of the present cases; for LACC that occurs in the supraglottic region, the extent of surgery varies according to the surrounding tissue invasion. Ideally, the tumor is completely removed during the first operation. However, as the structure of the head and neck is complex, at diagnosis, the disease is often at the advanced stage, and, at this point, the tumor has often spread along nerves. Thus, complete tumor removal is often difficult.^[3,9,20,21]^ In some cases, the ability to remove the entire tumor is impaired as the pathological examination of the margin is not reliable. Even in cases where the margin is negative, remote nerve spread can still be present.^[15]^

False-negative margin reports may lead to a clinician mistakenly believing that the tumor has been completely removed, which, in turn, affects the development of follow-up treatment schedule.^[19]^ Furthermore, preoperative evaluation of patients with cervical lymph nodes (cN)^+^ is feasible for cervical lymph node dissection, while for patients with cN^0^ it is not. This may be because cervical radiotherapy can also achieve the same effect of neck dissection.^[22]^ However, LACC is located in areas that are rich in lymph nodes, which is recommended for cervical lymph node dissection for the high rate of lymph node metastasis.^[23]^ For all of the cases in the present study, cervical lymph node dissection was performed.

It is generally believed that patients with positive margins, margins close to the tumor, or pathological findings of neurological involvement should be treated with radiotherapy or routine postoperative radiotherapy. Appropriate postoperative radiotherapy can improve the local control rate and tumor-free survival rate. Furthermore, preoperative radiotherapy can increase the tumor resection rate. The radiation field of radiotherapy should include the peritumoral nerve pathway because of neurological involvement. The cN^0^ patient may not include the neck area, but the neck-preventive irradiation may be feasible for tumors located in the rich lymph node site.^[24]^ The adequate radiotherapy dose is a prerequisite for the efficacy and should usually be greater than 60 Gy. Relapse is often observed if the dose for postoperative radiotherapy or radiotherapy is less than 60 Gy.^[25]^

It has been confirmed that adenoid cystic carcinoma often undergoes early neurological and hematogenous metastasis at early stages.^[2,3,13,26]^ Sometimes, metastasis can occur after many years of primary tumor treatment. Moukarbel et al^[27]^ reported, in a review of 15 cases, that the disease-specific survival rate was 69% and 49% at 5 and 10 years, respectively. In the present cases, the 5-year disease-specific survival rate was 100%. However, lung metastasis was observed in 1 patient 6 years after surgery. This indicates that the 5-year disease-specific survival rate is pretty high. However, distant metastasis may occur more than 5 years after treatment. Therefore, in cases of adenoid cystic carcinoma, long-term follow-up is required after treatment.

## 4 Conclusions

In conclusion, Although LACC is usually a slowly progressing cancer, late diagnosis can be fatal in some cases. Distant metastasis may still occur in patients with LACC even beyond 5 years after treatment. Therefore, patients with LACC require long-term surveillance.

## Acknowledgment

This manuscript has been edited and proofread by Medjaden Bioscience Limited.

## Author contributions

**Resources:** Lirong Bi.

**Supervision:** Le Sun.

**Writing – original draft:** Yu Cui.

**Writing – review & editing:** wang xin, Zhanpeng Zhu.
